# Feature Selection Combined with Neural Network Structure Optimization for HIV-1 Protease Cleavage Site Prediction

**DOI:** 10.1155/2015/263586

**Published:** 2015-04-15

**Authors:** Hui Liu, Xiaomiao Shi, Dongmei Guo, Zuowei Zhao, Yimin

**Affiliations:** ^1^Department of Biomedical Engineering, Dalian University of Technology, Dalian 116024, China; ^2^Second Affiliated Hospital, Dalian Medical University, Dalian 116027, China; ^3^Department of Advanced Medicine, Graduate School of Medicine, Hokkaido University, Kita 15, Nishi 7, Kita-ku, Sapporo, Hokkaido 060-8638, Japan

## Abstract

It is crucial to understand the specificity of HIV-1 protease for designing HIV-1 protease inhibitors. In this paper, a new feature selection method combined with neural network structure optimization is proposed to analyze the specificity of HIV-1 protease and find the important positions in an octapeptide that determined its cleavability. Two kinds of newly proposed features based on Amino Acid Index database plus traditional orthogonal encoding features are used in this paper, taking both physiochemical and sequence information into consideration. Results of feature selection prove that *p2*, *p1*, *p*1′, and *p*2′ are the most important positions. Two feature fusion methods are used in this paper: combination fusion and decision fusion aiming to get comprehensive feature representation and improve prediction performance. Decision fusion of subsets that getting after feature selection obtains excellent prediction performance, which proves feature selection combined with decision fusion is an effective and useful method for the task of HIV-1 protease cleavage site prediction. The results and analysis in this paper can provide useful instruction and help designing HIV-1 protease inhibitor in the future.

## 1. Background

Acquired immune deficiency syndrome (AIDS) is a severe disease which mostly causes patient's death during its terminal period. Most patients suffer from this disease because they are infected by HIV-1. Although many researches and investigations have been implemented, medicines or methods to entirely cure AIDS have not been found. However, there are some methods to relieve patient's ailment by medicines or therapies. HIV-1 protease inhibitor is such a kind of medicine that can be used to treat AIDS. HIV-1 protease is an enzyme which plays an important role in the replication progress. It cleaves proteins to smaller peptides, and these peptides are used to make up some important proteins that are essential for the replication of HIV-1 [1]. Thus inhibition of this protease is a reliable method to interfere the virus reproduction. HIV-1 protease inhibitor is a small molecule that can tightly bind to HIV-1 protease at the active cleavage sites, so that substrates which should normally be cleaved cannot bind to the protease. Normally, the protease binds with a protein in octapeptide length and cleaves it at the scissile bond. It is quite important to find which amino acid sequences can be cleaved, that is, the specificity of the protease. Also a good concept of which residues play more important roles in the cleavage progress is necessary. However, it is too costly and almost impossible to achieve these targets through experiments in laboratory. There are 20 amino acids in the natural world, so there can be 20^8^ kinds of octapeptides in all. It is impossible to test each octapeptide for confirming whether it can be cleaved by HIV-1 protease. Thus prediction of protease cleavage sites through computer programs becomes an economical and effective solution [2]. Machine learning methods can be used here to predict whether octapeptides are cleavable for the protease.

A lot of researches and investigations for HIV-1 protease cleavage sites prediction scheme have been carried out during the past two decades [3]. The previous investigations are mostly about the design of prediction models (classifiers) and methods of feature extraction [4]. Many classical classifiers, such as neural network, linear discriminant classifier, and support vector machine, are used in previous researches. In this paper, feed forward back-propagation (BP) neural network is used, which is a powerful learning system from the learning point of view. It is also succinct in structure and easy for programming. Researchers have also proposed many methods of feature extraction. Features here are mainly divided into two categories, features based on peptide sequence and features based on physicochemical properties. In this research, orthogonal encoding (OE) features based on peptide sequence, principal components analysis (PCA), and nonlinear Fisher transformation (NLF) based features which are extracted from Amino Acid Index (AAindex) database are fused to obtain comprehensive feature representation.

A typical HIV-1 protease cleavage sites prediction frame can be described as this: extract features from octapeptides, train a classifier based on the training samples, and then predict the label of a new unlabeled sample with the trained classifier. As the amount of information provided by a single kind of features is limited, the prediction accuracy will be faced with a bottleneck if only using a kind of features. Three kinds of original features are used in this paper, and experiments are carried out to test their classification performance. It is reasonable to fuse the three kinds of features as the input of classifier to improve classification accuracy [5]. Two fusion manners for features are used here: one way is used to train the classifier by combining the three kinds of features called combination fusion; the other one is used to train three classifiers separately with three kinds of features and then produce an output label based on the outputs of the three classifiers according to majority rule, which is called decision fusion. However, large feature space might result in overfitting of BP neural network and reduce classification performance of new samples [6]. In order to guarantee generalization capability of classifiers, features must be coordinated with the classifier.

According to statistical learning theory, the generalization capability of a classifier is determined by its Vapnik-Chervonenkis dimension [7]. If a BP network possesses a complex structure with too many nodes in the layers, it will face a quite high Vapnik-Chervonenkis dimension which will seriously reduce its generalization capability. Thus dimensionality reduction of feature space and optimization of network structure are needed [8]. Feature selection is an effective method for dimensionality reduction which is different from feature transformation. It retains the original structure of features and helps to understand the physical meaning in data [9]. Specific to our research, feature selection can help us find which positions and amino acid residues in an octapeptide play more important roles in deciding whether it can be cleaved. In our research, a feature selection method combined with structure optimization of neural network is used, which retains effective features and confirms neural network structure at the same time. Feature selection is conducted on the three kinds of features separately, and three subsets are got. Then the three subsets are fused in two different ways for testing, which were mentioned above.

In this paper, feature representation is spread by fusing three kinds of features to improve classification performance, and feature selection actually improves generalization capability of classifiers. Decision fusion based on three kinds of features in subsets gets excellent classification performance. The important positions and amino acid residues in peptides which demonstrated the cleavage specificity of HIV-1 protease are found. Our work can provide some instructive help for designing HIV-1 protease inhibitor.

## 2. Methods

### 2.1. Data Set

There are some classic data sets which have been collected and published. Cai and Chou [10] integrated several small data sets into generating the classic 362 set, which included 114 HIV-1 protease substrates assigned as positive samples and 248 non-HIV-1 protease substrates assigned as negative samples.

You et al. [11] got a relatively big data set from the published research results of HIV-1 protease specificity. They claimed the data set of 362 peptides was too small for investigating such a sophisticated issue [12]. That is why they built their data set of 746 peptides.

Kim and his colleague [13] collected 392 octapeptides added to the 362 data; thus a 754-sample data set was got, which included 395 HIV-1 substrates and 359 non-HIV-1 protease substrates.

Kontijevskis and his colleague [14] conducted a research that the collected data came from published research for relationship between protease and substrates from 1990 to 2005 and generated a big data set including 1625 octapeptides. There are 374 positive samples and 1251 negative samples.

In our research, these formerly used data sets are combined to enlarge the datasets and 3618 samples are got. After removing the contradictory and redundant samples, the dataset has 1922 octapeptides, which contains 596 positive samples and 1326 negative samples. This dataset is called 1922 dataset.

### 2.2. Feature Extraction

Numerous kinds of feature extraction methods for peptides have been proposed [15]. There are mainly two sorts of features that are usually extracted from a peptide: features based on peptide sequence and features based on physicochemical properties.

#### 2.2.1. Feature Extraction Based on Peptide Sequence

Feature extraction based on peptide sequence is a commonly used and classical method to represent a peptide for HIV-1 protease inhibitor prediction. Some methods to extract features are based on protein sequence, such as amino acid composition, n-order couple composition, pseudo-amino acid composition, and residue couple. However, these methods originally proposed to extract features of proteins, not particularly raised for peptide sequence. Usually a protein molecule is much larger than a peptide; thus a protein sequence contains much more structure information than a peptide. These methods cannot extract enough useful information from a small peptide molecule. Thus methods specially proposed for peptides are taken into consideration in our research. OE is one most frequently used method to employ a sparse representation. A 20-bit vector represents a kind of amino acid with 19 bits set to zero and one bit set to one. Each vector denoting an amino acid is orthogonal to the others. In this way, an amino acid sequence is mapped into a sparse orthogonal vector space. If a peptide sequence contains *M* consecutive amino acids, it can be represented by *M* × 20 features.

#### 2.2.2. Feature Extraction Based on Physicochemical Properties

Although OE features can provide good prediction accuracy for HIV-1 protease inhibitor, features just based on sequence cannot provide comprehensive feature representation. Features based on physicochemical properties of amino acids can provide different but quite useful information, which can effectively improve prediction accuracy. The inherently contained characteristics of amino acids can provide useful help for us to understand the specificity of HIV-1 protease [16].

The AAindex Database is a collection of amino acid indices in published papers [17]. An Amino Acid Index is a set of 20 numerical values representing any of the different physicochemical and biological properties of amino acids. The AAindex1 section of the AAindex Database is a collection of published indices together with the result of cluster analysis using the correlation coefficient as the distance between two indices. This section currently contains 544 indices.

Another important feature of amino acids that can be represented numerically is the similarity between amino acids. A similarity matrix which is called mutation matrix and it contains a set of 210 numerical values, 20 diagonal and 20 × 19/2 off-diagonal elements used for sequence alignments and similarity searches. The AAindex2 section of the AAindex Database is a collection of published amino acid mutation matrices together with the result of cluster analysis. This section currently contains 94 matrices.

Up to now, most methods of extracting features from peptides based on AAindex Database employ the amino acid indices. Many methods proposed for proteins can be used here, like autocorrelation function and pseudo amino acid composition. In our research, features extracted based on PCA and NLF of AAindex Database are used.

Nanni and Lumini utilize [18] all the amino acid indices in AAindex1 and the diagonals of the substitution matrices in AAindex2 and apply PCA and NLF to extract features from the original feature space. The two methods are introduced in the following part.

PCA based feature extraction is used to transform the original feature space into an orthogonal principal component space. The principal components are the *k* largest eigenvectors of the covariance matrix based on the original feature space. Here *k* is an undetermined integer. In this transformation, the first principal component has the largest possible variance, and each succeeding component in turn has the highest variance possible under the constraint that it be orthogonal to the preceding components. After conducting PCA to the original features, each kind of amino acid can be represented by 19 features.

NLF based feature extraction utilizes an objective function of the nonlinear Fisher transformation with the purpose of well separating patterns of different classes. 20 different labels can be put on the 20 kinds of amino acids. So discriminating the 20 amino acids becomes a supervised classification problem. The original Fisher transformation suffers from occlusion of neighboring classes, so the nonlinear Fisher transformation is proposed. After conducting NLF to the original features, each kind of amino acid is represented by an 18-feature vector.

In our research, three kinds of feature extraction methods are utilized: OE, PCA, and NLF based feature extraction methods considering they are specially proposed for peptide encoding. Experiments in the following part of this paper indicate that all the three sorts of features can provide good prediction performance.

### 2.3. Feature Selection

In a machine learning frame, dimensionality reduction is usually a highly important part which aims to reduce the classifier complexity and improve the classification accuracy. In some cases, both of the two aspects are taken into account, while sometimes one aspect is mainly focused on. There are two ways to implement dimensionality reduction: feature transformation and feature selection. Understanding the relationship and difference between them is very important. Feature transformation is carried out by mapping or combining features of the original feature space, a process that changes original features and generates new features. Feature selection is used to find the optimal (or suboptimal) feature subset from the original feature set and this process does not change the original features [19]. The choice of feature transformation or feature selection should be based on the specific problem for reducing dimensionality. In this paper, our research mainly focuses on the feature selection. There are three purposes for feature selection: to improve the classification accuracy of the classifier; to make classifier easier and faster, thus saving computing space and improving efficiency; to help us better understand the data generating process and the potential physical meaning in data. Sometimes feature selection is used not for improving classification accuracy but for simplifying the classifier and mining the potential physical meaning. Feature selection contains a number of aspects: definition of the objective function, feature sorting, searching criterion, and results verification. These methods for selecting feature subset are generally divided into three models: wrapper, filter, and embedded method. Wrapper is a black box integrating some kind of classifier to find the optimal (suboptimal) subset by verifying the classification accuracy of the selected feature subset. Filter is used to find the optimal (suboptimal) feature subset based on a certain criterion, independent of the choice of classifier, and is usually used in the pretreatment. Embedding method is used to accomplish feature selection while training the classifier with the training set; the specific method is different according to the different selected classifiers.

Feature selection is a relatively new problem in HIV-1 protease inhibitor prediction and it is the key thought in our research. It helps us find out the positions in octapeptides playing more important roles in deciding whether an octapeptide can be cleaved by HIV-1 protease. The important roles of amino acid residues that constitute octapeptides are also investigated. In our research, the feature selection task is separated into two steps: the preliminary step and the complete step. In the preliminary step, a wrapper feature selection method including a neural network is conducted and structure optimization of neural network is accomplished at the same time. In fact, feature selection combined with neural network optimization is conducted in previous research like CAFS [20]. However, CAFS meets great difficulty in dealing with high-dimension data. Specific to our task, it cannot select enough useful features for prediction. Therefore, a two-step scheme is conducted in our task. The special way to sort features will be explained in following part. In order to include enough useful features, relatively loose feature evaluation criteria are used in our method. An initial subset for the complete step is got when the preliminary step ends. In the complete step, the final subset is determined according to classification performance on validation samples based on the initial subset previously got.

A wrapper method is designed to provide better classification accuracy for the prediction task in the preliminary step. There is a neural network to examine the classification accuracy of different subsets in the feature selection algorithm; the useful and effective features can be selected for the following prediction process. It is important but difficult to determine the number of nodes in the hidden layer for a BP neural network. Too many nodes will cause high computational complexity and take up a lot of resources, while too few nodes cannot provide enough classification ability. Our method provides a solution to solve this problem. It is used to choose effective features from the data set, which shows us the more important positions in octapeptides and more important amino acid residues at different positions indicating the HIV-1 protease specificity.

#### 2.3.1. Feature Selection Combined with Neural Network Structure Optimization

The following is the feature selection scheme for finding the useful features and accomplishing with network structure optimization at the same time. One severe drawback of neural network is that its optimal structure is not explicit. A structure optimized neural network can provide reliable classification ability and guarantee good generalization capability. Therefore, feature selection is conducted with neural network structure optimized feature in this paper. The feature selection scheme is divided into two steps. The preliminary selection with network optimization accomplishes the initial selection of useful features. The complete selection confirms the final subset and network structure.

An octapeptide is denoted by *p*4*p*3*p*2*p*1*p*1′*p*2′*p*3′*p*4′, where *p* represents a kind of amino acid residue. The scissile bond that may be cut by HIV-1 protease is between *p*1 and *p*1′. Each position of an octapeptide can be expressed by a group of features as mentioned above: OE, PCA based features, and NLF based features. It is supposed that if one position is nearer to the scissile bond than others, it plays more important role in manifesting HIV-1 protease cleavage specificity. Thus, a special feature sorting criterion is used to rearrange features and place particular emphasis on middle positions. Symmetrical positions, for example, *p*1 and *p*1′, are considered of equivalent importance. The correlation of different features is also taken into consideration, and features are rearranged with two steps. Firstly, it arrays the sequence as *p*1*p*1′*p*2*p*2′*p*3*p*3′*p*4*p*4′. Features with smaller correlation values are more important than features with larger correlation values. Then it sorts the features at each pair of symmetrical positions from small correlation value to large correlation in step two. For example, correlation values of features at *p*1 and *p*1′ are computed, and then features are sorted based on them. Repeat the same operation on *p*2 and *p*2′ and so on. In this paper, Pearson product-moment correlation coefficient is shown in

(1) rij=∑pxi−x−ixj−x−j∑pxi−x−i2∑pxj−x−j2, i=1,2,…,p.

*r*_*ij*_ stands for the correlation value between features *i* and *j*. *x*_*i*_ and *x*_*j*_ stand for the value of features *i* and *j*; ${\overset{-}{x}}_{i}$ and ${\overset{-}{x}}_{j}$ stand for the mean value of features *i* and *j* based on *p* samples, respectively. After all of the correlation values for each possible feature combination are computed, correlation of each feature is computed according to

(2) $$\begin{array}{c} {\text{cor}}_{i}=\frac{\sum_{j=1}^{M}\left|r_{ij}\right|}{\left(M-1\right)},\;\text{here}\;i\neq j, \end{array}$$

where cor_*i*_ is the correlation for feature *i* and *M* is the number of features used here.

**Figure 1:**
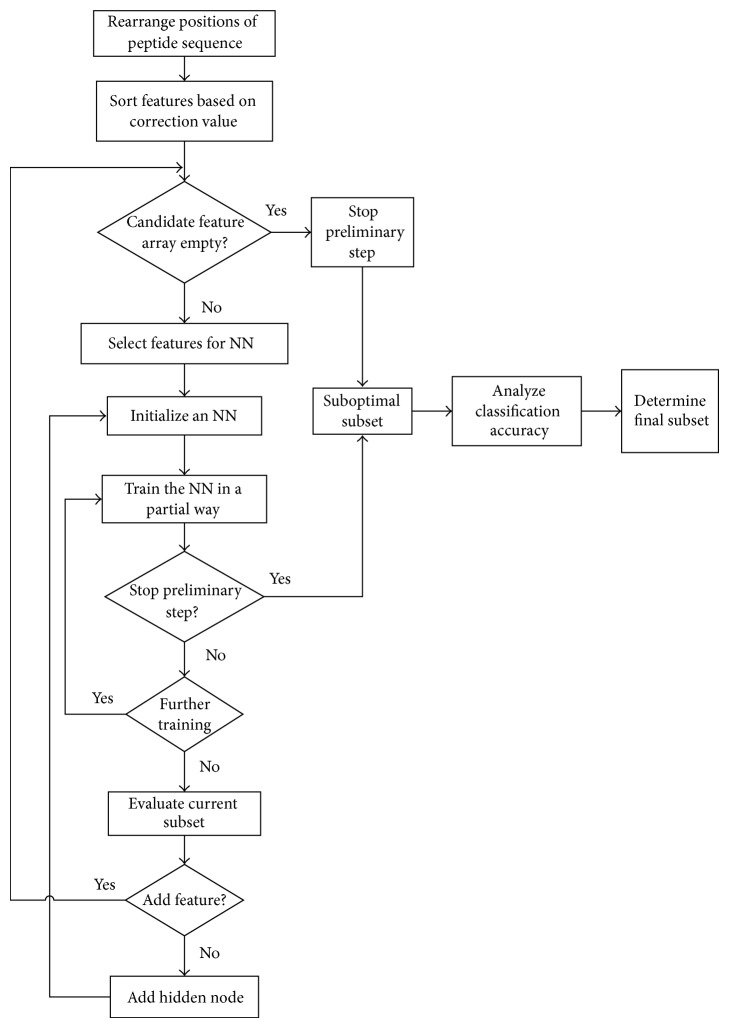
Flowchart of the feature selection method. In the flowchart, a subset which is shown as suboptimal subset is got after the preliminary step. The part before the suboptimal subset is got can be referred to as the preliminary step. Then a final subset is got according to the classification accuracy based on this subset in the complete step. The part after the suboptimal is got can be referred to as the complete step.

For the task of feature selection, data samples are randomly divided into two groups: training set and validation set. Training set is used to train neural network; validation set is used to evaluate the performance of feature subsets and guarantee the generalization capability of neural network in feature selection progress. The sample proportion of training set and validation set in all samples is 0.6 and 0.4, respectively. The general information of the feature selection method is shown in Figure 1, and the details are in the following part.

**Step 1 .**

An octapeptide is denoted by *p*4*p*3*p*2*p*1*p*1′*p*2′*p*3′*p*4′, where *p* represents a kind of amino acid residue. Features are initially extracted with different methods according to this original sort. The positions nearer to the scissile bond are supposed to play more important roles, and symmetrical positions are equally important. An octapeptide will be cleaved at the scissile bond if it is cleavable. The experimental results prove our hypothesis, so the sequence of octapeptide is rearranged to *p*1*p*1′*p*2*p*2′*p*3*p*3′*p*4*p*4′.

**Step 2 .**

Sort the features at each pair of symmetrical positions from small correlation value to larger one. Thus a new feature array is got from which features are added to subset during feature selection. This feature array is called candidate feature array. When a feature is added to the subset, correspondingly it is eliminated from the candidate feature array. Generally, an initial subset is needed as a start in feature selection and the initial subset is the first feature in the candidate feature array.

**Step 3 .**

Judge if the feature selection should stop by examining whether the candidate feature array is empty. If it is empty, the algorithm will stop. This is termination criterion 1 in this algorithm. After the algorithm stops, the current feature subset is the final subset in preliminary step and the initial subset for the complete step.

**Step 4 .**

Feature selection starts from the first feature in the candidate array. Feature is picked out in order from this array and temporarily added to the current subset with a so-called temporary subset created. If the temporarily added feature is evaluated usefully in Step 9, this feature will finally be added to the current subset, which means the temporary subset will become the new subset. Otherwise, this feature will be eliminated from the candidate array and the temporary subset is cancelled. The subset will still be the original one.

**Step 5 .**

Initialize a BP neural network. The number of input layer nodes is the size of temporary subset and the number of hidden layer nodes is initially set to one. In the following process, this number may be increased as needed. The number of output layer nodes is set to one. The true label of a sample in our data is 0 or 1, so the outputs of network are real numbers close to 0 or 1 after training.

**Step 6 .**

Train the network in a partial way which means training the network *τ* epochs each time. Here *τ* epochs can be called a training series. The network is trained *τ* epochs in each time whether it is convergent or not; the training error is saved. After every training series, a partially trained network is got, and the samples in validation set are classified. The classification accuracy and validation error are got and saved. To classify the samples in validation set a threshold between 0 and 1 is needed for label determination.

**Step 7 .**

Check termination criterion 2 after every partial training series. Here criterion 2 is tested according to the validation error, and validation error has been achieved in Step 6. All validation errors after partial training are saved and compose a validation error array. If there is a *T* time's successive increase for validation error exceeding a threshold *λ*, it means the network is overfitting; thus the algorithm should stop. After the algorithm stopping, the current feature subset is the final subset for preliminary step and the initial subset for complete step.

**Step 8 .**

Determine whether further training is needed. If the difference between training errors of current training series and previous training series is smaller than a specified threshold *ε*, it means the network is convergent and the training should stop. Step 6 mentions that a classification accuracy value on validation set is got after each training series, so when the network training is finally over, an array of classification accuracy is got which will be used in Step 9.

**Step 9 .**

Evaluate the temporary subset after adding a new feature by analyzing the classification accuracy array. If there is a significant ascending trend in this array, it means that the temporarily added feature makes sense, and this feature provides useful information and improves classification accuracy. Otherwise go to Step 10. As adding a feature does not improve classification performance on validation samples, it may be due to the fact that current network does not contain enough hidden nodes. Thus adding a hidden node becomes a considerable solution.

**Step 10 .**

Determine whether a node in hidden layer should be added. Firstly, we temporarily add a hidden node for the network and train with the temporary subset again. If there is a significant ascending trend in classification accuracy array this time, it means that adding a hidden node can effectively improve classification performance. Thus the feature picked out in Step 4 is finally added to the subset, and a hidden node is added in the network. If adding a hidden still cannot improve classification performance, the feature picked out at Step 4 is regarded as useless and will be eliminated from the candidate feature array, and the newly added hidden node will be eliminated too.

**Step 11 .**

After the preliminary step, an initial subset is got. Actually, a group of subsets are got to be analyzed from the initial subset. During the preliminary selection, a new feature is successfully added to the subset for each time, classification accuracy of validation set is got and saved, and the corresponding current node number of hidden layers is also saved. Thus each subset corresponds to a validation accuracy and hidden node number. This validation accuracy array is analyzed and helps determining the final subset.

**Step 12 .**

In the end, the final subset will be determined based on the results achieved in Step 11. When the classification accuracy on validation set is at a relatively high and steady level even if it is not the largest value, the subset still includes enough effective features and contains enough useful information. According to this criterion, the final subset is determined expecting it will provide good classification performance. Meanwhile, the corresponding network structure is confirmed which means the node numbers of input layers and hidden layers are determined.

#### 2.3.2. Determining the Final Subset

After the preliminary selection, a subset with some redundant features is got. As features in the subset are added one by one during preliminary selection, classification accuracy on validation samples is got and saved after a new feature is added. Meanwhile, the number of hidden nodes corresponding to each subset is also saved. The reason for carrying on two steps is to include enough useful features. So a loose feature evaluation criterion is conducted in preliminary selection. However, this manner will also contain some redundant features, so the complete step is needed to remove them. In fact, when the classification accuracy of validation set has a relatively high and steady level even if it is not the largest value, the subset still includes enough effective features and useful information. In the following part, this method to determine the final subset according to the different validation classification performance of subsets is introduced. This work uses the three subsets got based on three kinds of features after preliminary selection and it is previously mentioned.

**Figure 2:**
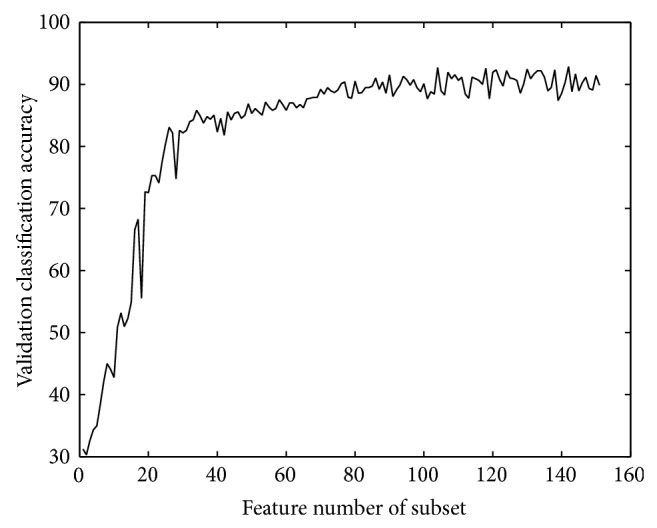
Validation accuracy of OE features.

*Orthogonal Encoding Based Features*. The classification accuracy on validation set during preliminary selection for OE features is shown in Figure 2.

A subset of 151 features is got after the preliminary selection. When the subset contains 142 features, the classification accuracy on validation set gets largest value which is 92.8479. However, when the subset contains 90 features, validation classification accuracy already obtains a relatively high value. But the 90-feature subset is not the final subset. The final subset should have a steadily high classification accuracy level avoiding too many ups and downs. The final subset should not miss too many useful features so as to keep good classification performance and include as few as redundant features. Thus the final subset is the 104-feature one. The numbers of features distributed at 8 positions are 0, 16, 17, 19, 19, 17, 16, and 0. It can be easily found most features distributed at *p*1, *p*1′, *p*2, *p*2′, and *p*3, *p*3′. There is a redundant feature which is inherently produced in OE. The redundant feature is removed after the two-step feature selection at most positions. A neural network is got after feature selection, containing 12 nodes in hidden layer.

**Figure 3:**
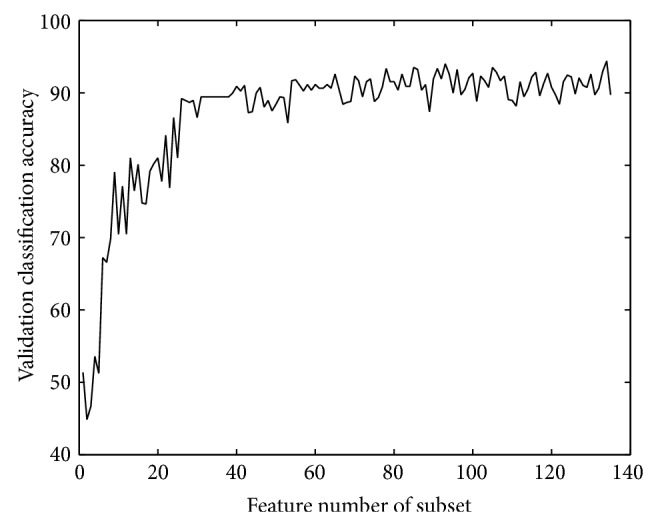
Validation accuracy of PCA features.

*PCA Based Features*. The classification accuracy on validation set during preliminary selection for PCA based features is shown in Figure 3.

After the preliminary selection, a subset that contains 135 features is got. The largest value of classification accuracy based on validation set is 94.4083. Nevertheless, when the feature subset contains 134 features, the classification accuracy of validation set gets a local maximum value. The final subset is determined by following the same method mentioned above in OE features and the final choice is the 102-feature subset. The numbers of features distributed at 8 positions are 0, 8, 19, 19, 19, 18, 8, and 0. A neural network is got after feature selection, containing 12 nodes in hidden layer.

**Figure 4:**
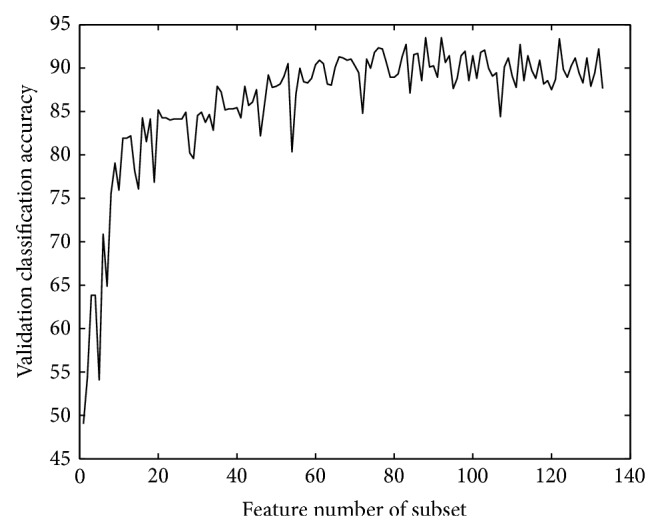
Validation accuracy of NLF features.

*NLF Based Features*. The classification accuracy on validation set during preliminary selection for NLF based features is shown in Figure 4.

The preliminary selection produces a 133-feature subset. When the subset contains 88 features, the classification accuracy of validation set gets the largest value of 93.4980, and the 88-feature subset bears a local maximum value for validation set classification accuracy. If more features are added, the classification accuracy relatively stays high. Thus, the final subset is the 103-feature one after the complete selection. The numbers of features distributed at 8 positions are 0, 8, 13, 17, 16, 15, 7, and 0. A neural network is got after feature selection, containing 13 nodes in hidden layer.

## 3. Results and Discussion

Sufficient experiments are conducted to compare the performance of final subsets that we get, the fused subsets, the original features, and fused original features using 10-fold cross validation. Tenfold cross validation is a widely used method to examine classification performance. Four parameters, accuracy, sensitivity, specificity, and Matthews Correlation Coefficient (MCC) [21], are calculated based on the results of 10-fold cross validation experiments to evaluate classification performance. The 10-fold validation experiments are conducted in a way as follows: all samples are divided into 10 parts, while each part is used as test set and other parts are used as training set by turns; thus every sample gets a chance to be test and it will be assigned a label according to the classifier output; by comparing the original label and network outputting label of samples, the four evaluation parameters of accuracy, sensitivity, specificity, and Matthews Correlation Coefficient are computed. In the following parts, the results of the three kinds of original features, three subsets after feature selection, and features with fused methods will be discussed and analyzed.

### 3.1. Three Kinds of Original Features Separate and Feature Fusion

A single kind of features containing the information is limited, so the prediction accuracy will be faced with a bottleneck by only using a single kind of features. Although OE features might provide massive information, only sequence information is not enough. Thus features based on physiochemical properties are taken into consideration, and PCA and NLF based features are used in this paper. They contain different information, respectively, and quite differ from OE features. Two kinds of methods for feature fusion are used to improve classification accuracy: combination fusion and decision fusion. Combination fusion is used to train the classifier by combining the three kinds of features, and decision fusion is used to train three classifiers separately with the three kinds of features and produce an output label based on the outputs of the three classifiers according to majority rule. Experiments are conducted to examine the classification performance based on the three kinds of original features plus the fused features. The results are shown in Table 1.

Table 1 shows the results of 10-fold cross validation for each kind of original features and fusion method. Accuracy, sensitivity, specificity, and MCC are calculated to examine the prediction performance. The feature number of the three kinds of original features is shown in Table 1. The feature number of combination fusion is got by summing the feature number of the three kinds of original features. The feature number of decision fusion is not necessary to calculate, as it is different from combination fusion. The sizes of original feature space for OE, PCA, and NLF based features are 160, 152, and 144, respectively. The size of feature space for combination fusion is 456 which is a high value for a prediction task, while the size of feature space for decision fusion cannot be calculated by simple addition operation. According to Table 1, all the three kinds of original features get good classification performance, and OE features gain the best performance. PCA and NLF based features get close performance, which provide different information and are complementary in the following feature fusion scheme. The two feature fusion methods get obviously different results: decision fusion exceeds combination fusion substantially. Combination fusion of the three kinds of features is inferior to OE features but close to PCA and NLF based features, while decision fusion of the three kinds of features gets superior performance to all of them. The reason for combination fusion with larger feature space not obtaining better performance might be that too large feature space results in overfitting for BP neural network and reduces generalization capability for predicting new samples. Due to the high dimensionality of feature space, the Vapnik-Chervonenkis dimension is severely high and causes overfitting; thus the advantage of fusing multiple kinds of features does not show. In a word, overfitting worsens the generalization capability of the network. The reason for decision fusion getting an excellent result might be that there are three networks trained by the three kinds of features, respectively, and the networks perfectly deal with the features. Three different kinds of information are effectively used. There is not too high-dimension feature space for each network which guarantees the generalization capability. Meanwhile, the voting mechanism uses the three kinds of information sufficiently.

### 3.2. Feature Selection and Feature Fusion

Known from the result of combination fusion, there is overfitting in the trained network. To find the specificity of HIV-1 protease and solve overfitting of network, feature selection is conducted. Feature selection can find the most useful features that indicate which positions and amino acid residues play more important roles in demonstrating the specificity of HIV-1 protease and can simultaneously accomplish network structure optimization. Feature selection is firstly conducted on the three kinds of original features in this paper. The importance of different positions is confirmed according to the number of features retained at each position after feature selection: the more features a position contains, the more important it is.

**Figure 5:**
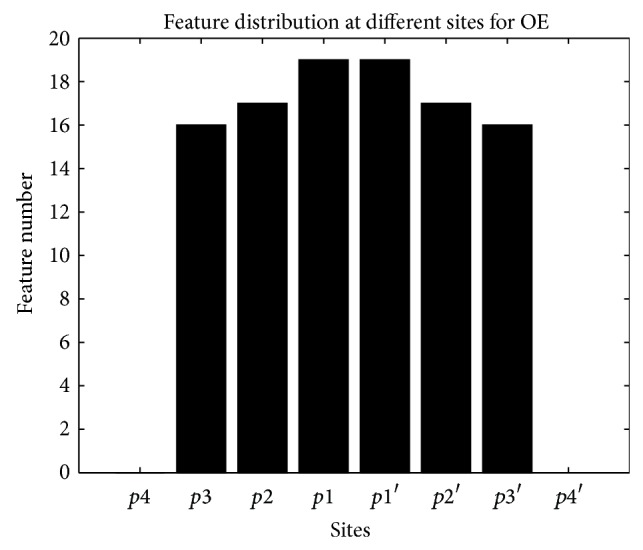
Feature number at different sites after feature selection for OE features.

The distribution of features at different sites for OE features is shown in Figure 5. The distribution of features retained at the 8 positions is apparent: there are 0, 16, 17, 19, 19, 17, 16, and 0 features at *p*4, *p*3, *p*2, *p*1, *p*1′, *p*2′, *p*3′, and *p*4′, respectively. *p*4 and *p*4′ do not retain any feature after feature selection. The four positions nearer to the scissile bonds *p*2, *p*1, *p*1′, and *p*2′ contain the most features in the subset. This result proves our supposition that the positions nearer to the scissile bond play more roles in determining whether an octapeptide is cleavable. The size of feature space is reduced from 142 to 104, which is large reduction ratio.

**Figure 6:**
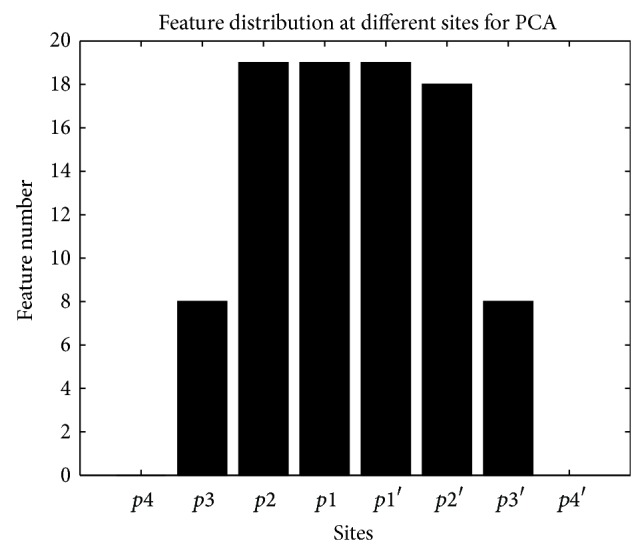
Feature number at different sites after feature selection for PCA features.

The distribution of features at different sites for PCA features is shown in Figure 6. There are 0, 8, 19, 19, 19, 18, 8, and 0 features at *p*4, *p*3, *p*2, *p*1, *p*1′, *p*2′, *p*3′, and *p*4′, respectively. There is no feature retained at *p*4 and *p*4′, and *p*2, *p*1, *p*1′, and *p*2′ contain the most features. This result also proves our supposition. The size of feature space is reduced from 135 to 91.

**Figure 7:**
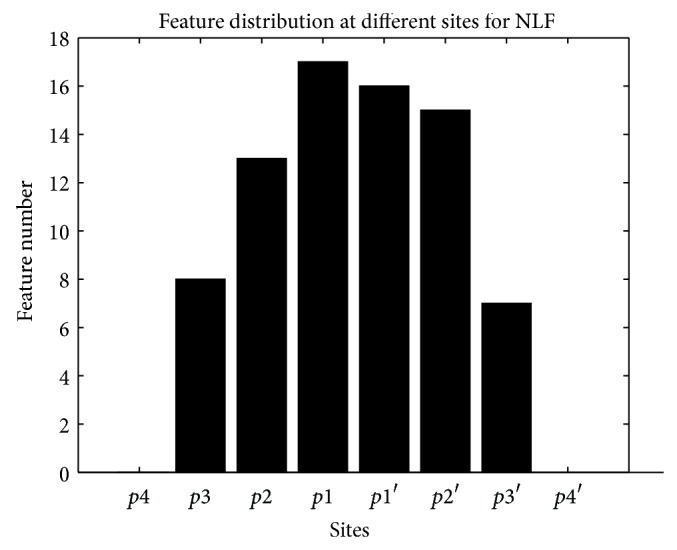
Feature number at different sites after feature selection for NLF features.

The distribution of features at different sites for NLF features is shown in Figure 7. There are 0, 8, 13, 17, 16, 15, 7, and 0 features at *p*4, *p*3, *p*2, *p*1, *p*1′, *p*2′, *p*3′, and *p*4′, respectively. Still there is no feature retained at *p*4 and *p*4′, and *p*2, *p*1, *p*1′, and *p*2′ contain the most features. This result proves our supposition again. The size of feature space is reduced from 133 to 76, which is a quite large reduction ratio.

**Figure 8:**
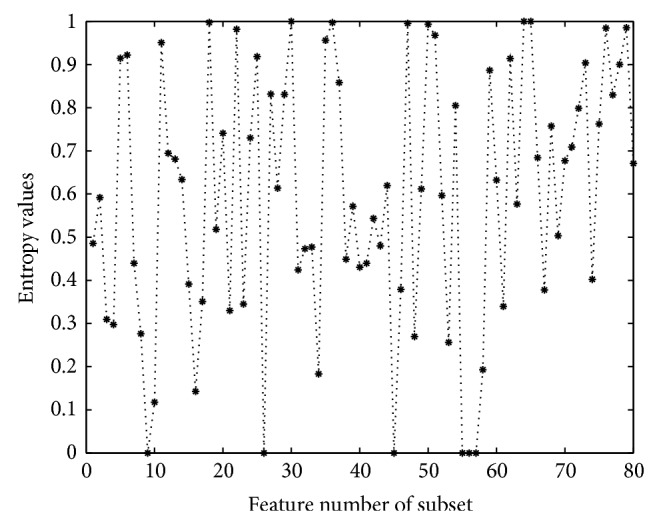
Entropy values of each feature in OE subset. Each asterisk represents the entropy value of each feature. And their corresponding values are shown according to the order they are added to the subset.

The statistical results of the feature distribution at 8 positions after feature selection and the three histograms perfectly prove our supposition about the importance of positions nearer to the scissile bond. On the other hand, the biological meanings of chosen features can be estimated by analyzing the statistical results of samples in dataset. Each feature in the subset of OE features represents one kind of amino acid residue; thus computing the entropy values of all chosen features based on the statistical results of subset will prove the effectiveness of chosen features.  Figure 8 shows the entropy values of chosen features in OE subset.

As shown in Figure 8, features at *p*1 and *p*1′ are first added in the subset at the beginning. The earlier added features obtain smaller entropy values than the following ones, which mean the earlier ones are more relevant to the samples judging from single feature. Some of them get zero values, and it means that the amino acid residues corresponding to these features are much relevant to the samples, thus proving the validity of our work. The following added features from *p*1 and *p*1′ obtain larger values, and it means these features are less relevant to the samples for the perspective of individual feature. Though these features are less relevant to the samples, it does not mean they are useless for the prediction task. The entropy value of feature does not take the dependence of features into consideration, while features with large entropy values may obtain good prediction ability combining with other features. Therefore, these features are still important for our research. Following features in *p*1 and *p*1′, features in *p*2, and *p*2′ are added to the subset. Still the earlier added features obtain smaller entropy values and have more relevance to the samples; the following features have larger entropy values. The same conclusion with the previous one will be got for the features in *p*2 and *p*2′. The prediction ability of chosen features is evaluated in the following part, and it turns out to be very good. As BP neural network can simultaneously deal with many input features, instead of dealing with single feature, interdependence between input features is taken into consideration. Thus a single feature with larger entropy value which combined with other features may get good prediction capability. At last, features in *p*3 and *p*3′ are added to the subset; their entropy values are relatively higher than the values of *p*1, *p*1′, *p*2, and *p*2′ which means features in more far positions from the scissile bond are less important for the specificity of HIV-1 protease.  Figure 8 demonstrates the chosen features in the subset are effective and the nearer positions to the scissile bond are more important.

After feature selection for three kinds of original features, three subsets are got. In order to improve prediction performance, we test the classification performance on the three subsets and use two fusion methods to apply fused features of the subsets.  Table 2 shows the results of 10-fold cross validation experiments.

Table 2 shows the results of 10-fold cross validation after feature selection for each kind of features and fusion method. Accuracy, sensitivity, specificity, and MCC are calculated to examine the prediction performance. The feature number of the three kinds of features after feature selection is shown in Table 2. The feature number of combination fusion is got by summing the feature number of the three subsets after feature selection. The feature reduction ratio is calculated by dividing the feature number of subset with original feature number. The feature number of decision fusion is not necessary to calculate, as it is different from combination fusion. The results in Table 2 show that three subsets still gain good classification performance with large feature reduction ratio after feature selection, and they are very close to the performance of original features correspondingly. This means the three subsets successfully retain the useful and effective features which provide meaningful information. The performance of combination fusion has been improvement in the three subsets, and it is better than the result of original PCA and NLF based features and combination fusion of three kinds of original features. The result of combination fusion based on three subsets proves the useful features are retained in the reduced feature space insuring high accuracy of classification and redundant features are eliminated avoiding over fitting. Decision fusion of three subsets gets excellent performance better than combination fusion based on three kinds of original features, and it is the best result in all of the experiments. It means that feature selection simplifies the network and improves generalization capability. Decision fusion sufficiently makes use of the different kinds of information contained based on the three kinds of features, and it produces a wonderful prediction performance. Decision fusion after feature selection is a good solution for HIV-1 protease inhibitor prediction and can provide help for HIV-1 protease inhibitor design in future.

## 4. Conclusions

Understanding the specificity of HIV-1 protease can help human beings design effective protease inhibitor to treat AIDS. Judging whether a peptide can be cleaved by HIV-1 protease is the key point, and machine learning is an economical solution for solving this problem. To get comprehensive feature representation, three kinds of features are extracted from peptide sequences in this paper. However, large feature space causes overfitting of neural network. In order to guarantee the generalization capability, a two-step feature selection is conducted to eliminate the redundant features and reserve the useful features. Feature selection also helps us to understand the specificity of HIV-1 protease. The positions nearer to the scissile bond are supposed to play more important roles, and the results of feature selection prove this supposition. In fact, all the features at *p*4 and *p*4′ are eliminated after feature selection. To improve prediction accuracy, two kinds of feature fusion methods are used. Combination fusion is proved not suitable here, while decision fusion improves prediction performance greatly. Thus feature selection combined with decision fusion is a good solution for HIV-1 protease cleavage site prediction. Our work can provide help for designing HIV-1 protease inhibitor. In the future, more sufficient feature selection method and effective classification model are expected to solve this task perfectly.

## Supplementary Material

The document covers the design of the feature selection method combined with neural network structure optimization. Meanwhile, this document contains the feature extraction of traditional orthogonal encoding features, PCA and NLF based features. Combination fusion and decision fusion method based on these features aim to get comprehensive feature representation and improve prediction performance are listed in the document.

## Figures and Tables

**Table 1:** Classification results of original features.

Reduced features	Accuracy	Sensitivity	Specificity	MCC	Feature number
OE	0.9214	0.9011	0.9351	0.8198	160
PCA	0.9162	0.8977	0.9253	0.8083	152
NLF	0.9136	0.9077	0.9223	0.8029	144
Combination fusion	0.9147	0.8943	0.9238	0.8048	456
Decision fusion	0.9344	0.9245	0.9449	0.8501	

**Table 2:** Classification results of reduced features.

Reduced features	Accuracy	Sensitivity	Specificity	MCC	Feature number	Feature reduction ratio
OE	0.9214	0.8993	0.9329	0.8191	94	0.4125
PCA	0.9110	0.9044	0.9178	0.7992	91	0.4013
NLF	0.9110	0.8775	0.9306	0.7934	74	0.4861
Combination fusion	0.9199	0.8977	0.9299	0.8161	259	0.4320
Decision fusion	0.9355	0.9211	0.9442	0.8518		
